# Building Resilience During COVID-19: Recommendations for Adapting the DREAM Program – Live Edition to an Online-Live Hybrid Model for In-Person and Virtual Classrooms

**DOI:** 10.3389/fpsyg.2021.647420

**Published:** 2021-07-12

**Authors:** Julia Parrott, Laura L. Armstrong, Emmalyne Watt, Robert Fabes, Breanna Timlin

**Affiliations:** Department of Human Sciences, DREAM Lab/Child Meaning and Resilience Laboratory, Saint Paul University, Ottawa, ON, Canada

**Keywords:** resilience, COVID-19, children, mental health promotion, mental illness prevention

## Abstract

In standard times, approximately 20% of children and youth experience significant emotional, behavioral, or social challenges. During COVID-19, however, over *half* of parents have reported mental health symptoms in their children. Specifically, depressive symptoms, anxiety, contamination obsessions, family well-being challenges, and behavioral concerns have emerged globally for children during the pandemic. Without treatment or prevention, such concerns may hinder positive development, personal life trajectory, academic success, and inhibit children from meeting their potential. A school-based resiliency program for children (DREAM) for children was developed, and the goal of this study was to collaborate with stakeholders to translate it into an online-live hybrid. Our team developed a methodology to do this based on Knowledge Translation-Integration (KTI), which incorporates stakeholder engagement throughout the entire research to action process. KTI aims to ensure that programs are acceptable, sustainable, feasible, and credible. Through collaboration with parents and school board members, qualitative themes of concerns, recommendations and validation were established, aiding in meaningful online-live translation. Even though the original program was developed for intellectually gifted children, who are at greater risk for mental health concerns, stakeholders suggested using the program for both gifted and non-gifted children, given the universal applicability of the tools, particularly during this pandemic time period when mental health promotion is most relevant. An online-live approach would allow students studying at home and those studying in the classroom to participate in the program. Broader implications of this study include critical recommendations for the development of both online-live school programs in general, as well as social-emotional literacy programs for children.

## Introduction

In regular times, approximately 20% of children and youth experience significant emotional, behavioral, or social challenges (Liratni and Pry, 2011; Kroesbergen et al., 2016; Pilarinos and Solomon, 2017). However, during the COVID-19 pandemic, over *half* of parents have reported significant mental health symptoms in their children, and nearly everyone reports some symptoms (Kar et al., 2020; Shah et al., 2020). Specifically, depressive symptoms, anxiety, contamination obsessions, family well-being challenges, and behavioral concerns have emerged globally for children during the pandemic (Fegert et al., 2020). Without treatment or prevention, such concerns may hinder positive development, personal life trajectory, academic success and inhibit children from meeting their potential (Pilarinos and Solomon, 2017). Therefore, certain provincial governments (e.g., Quebec) have recommended that a “kit be created that is aimed at parents, teachers, adolescents and children on emotional management in times of confinement and deconfinement”^1^. The current research aims to provide such a child well-being toolkit, adapted from our existing live version of our program to an online-live hybrid that could be administered in regular classrooms and virtual classrooms. Ultimately, our program’s goal is to promote resilience and meaning, as well as address child mental health needs during this challenging time.

### How Might a Social-Emotional Literacy (SEL) Program Aimed at Building a Meaning-Mindset Address Children’s Needs?

Social-Emotional Literacy programs’ designs provide specific skills and tools aimed to help them cope with destructive events (Fraser, 2011). Such skills could help children adjust to this unique time and aid in social and academic success (Peterson and Ray, 2006). Particularly during challenging times, finding a sense of meaning and purpose, a “hero’s journey,” or channeling the negative into positive change becomes relevant (Wong, 2020). Traditional SEL programs do not focus on the concept of meaning, and this is a gap in existing mental health promotion programming for children. Therefore, combining an SEL program with one that aims to cultivate a *meaning-mindset* might help children learn and grow in the face of the current challenging experiences (Dweck, 2016; Wong, 2017).

For children, a meaning-mindset can be cultivated in the following ways (Frankl, 1986; Armstrong, 2016; Wong, 2017; Armstrong et al., 2019):

• Believing in their own ability and skills to challenge unhelpful thoughts or attitudes, problem-solve, and take a healthy, realistic stance toward challenges.
• When faced with difficult feelings, viewing these feelings as an alarm bell indicating that they can take helpful action to regulate these feelings.
• Helping others, volunteering, and giving to or creating something for others.
• Developing and maintaining positive social connections (e.g., secure, supportive relationships with adults and peers) and feeling valued by others.
• Being regularly involved in valued activities (e.g., sports, music, or other extracurricular activities) that they look forward to would have difficulty giving up and perceive as “fun.”
• Having curiosity and openness to learning and other new experiences.
• Experiencing meaningful moments (e.g., experiencing nature, being excited by learning, noticing everyday joys) and expressing gratitude or appreciation for everyday experiences.
• Maintaining hope, even in the face of difficulties.

We have developed an SEL program that aims to foster a meaning-mindset called DREAM – Developing Resilience through Emotions, Attitudes, and Meaning.

### Developing Resilience Through Emotions, Attitudes, and Meaning (DREAM)

To address children’s specific needs, incorporating the thoughts, concerns, and opinions of children and their circle of care—educators, parents, school board members—is a natural progression for improving programs for this demographic. The DREAM Program has two main goals: the first is to support the community, including the children, their families, and schools, with an evidence-based sustainable toolkit, giving children skills they can use throughout their life. The second goal is to increase a sense of meaning, social skills, and agency over thoughts and behaviors to improve overall mental health (Armstrong et al., 2020). “Meaning” (resilience) is defined in this program as the cultivation of a meaning-mindset. Meaning is a protective factor for mental illness (Frankl, 1986; Armstrong, 2017; Wong, 2017). A meaning-mindset may help children understand the challenges faced during this time and allow them to cope and thrive, leading to long-term well-being (Wong, 2017). Second Wave Positive Psychology (PP2.0) is the theoretical foundation associated with a meaning-mindset and is the foundation of DREAM (Armstrong et al., 2020). PP2.0 was chosen because it focuses on social connectedness, values, and ideals as goals to work-toward (Wong, 2011).

One PP2.0 theory is called REAL (Rational Emotive Attachment Logotherapy; Armstrong, 2016, 2017). REAL works to target three primary sources of suffering: poor attachment schemas, meaninglessness, and irrational thoughts. REAL addresses these problems in the social context by building attachment bonds using play and social-emotional literacy skills. It also engages children in meaningful activities and meaning-oriented thinking, which can lead to a perspective change and recognition of meaning in the moment. Further, REAL uses rational thinking tools that value the good and bad parts of life, along with the meaning that comes from them (Armstrong, 2016). Through this theoretical framework, the “live” (original) version of DREAM, administered by a team of clinicians rather than online, enhanced resilience, measured as children’s hope for the future, positive self-concept, agency over thoughts and behaviors, social/emotional literacy, openness to learning, community engagement, and new experiences (Armstrong et al., 2020). It also significantly reduced internalizing and externalizing mental health symptoms (Armstrong et al., 2020). We collaborated with key stakeholders to develop an approach that would allow for flexible delivery to increase the program’s reach and sustainability. The virtual classrooms that were introduced at the beginning of the pandemic affirmed our exploration of flexible delivery styles as clinicians were unable to be in the classroom to facilitate the program.

### Knowledge Translation and Community Collaboration

Knowledge translation is a foundation of constructivism and is used to create a program that meets its intended users’ needs. Armstrong (2009, 2017), Armstrong et al. (2020) developed the Knowledge Translation-Integration (KTI) framework, which set the groundwork for the development of the DREAM Program. The KTI approach addresses the research to action gap by engaging stakeholders throughout the program development, evaluation process and by focusing on the program’s sustainability, accessibility, feasibility, and credibility (Armstrong, 2017). To be *feasible*, the program must be perceived as easy to implement. To be *acceptable*, the program must integrate relevant research and stakeholder’s needs (Armstrong, 2017). To be *credible*, the program must have face validity and appear to achieve the desired outcomes that address stakeholders’ needs and, once developed, it must be found to meet those needs (Armstrong, 2017). To be *sustainable*, the program must demonstrate that the long-term targets are maintained, and the program can be locally administered in an ongoing manner without the need for researcher involvement, which ties together the four blocks of KTI (Armstrong, 2017). Through the KTI framework, the needs of stakeholders are identified and are used as goals for the DREAM program development. The KTI framework underlines the program’s success because it ensures the program is meeting the needs of the stakeholders in the short and long term.

### Stakeholder Consultation

Due to the nature of KTI, stakeholder consultation is weaved throughout the development, implementation, and evaluation of DREAM. The literature has some key recommendations for stakeholder engagement within education systems and with families in order to maximize its potential. Specifically, facilitators of stakeholder engagement involve regular meetings, clear responsibilities, using plain language, and using a variety of approaches (i.e., focus groups, interviews, and questionnaires; Camden et al., 2015). Barriers to stakeholder engagement include limited time and resources, technology proficiency, schools’ institutional nature, and access to technology (Camden et al., 2015; Olofsson et al., 2015). Researchers found that when a variety of methods are used to engage stakeholders, more robust results are gathered (Albrecht et al., 2017).

Social-Emotional Literacy programs are most effective when the learning happens in school, at home, and in the community, and all relevant stakeholders are involved throughout the development and implementation stages (Weissberg and O’Brien, 2004). Consultation with school boards is necessary for the development of a child-targeted SEL program (Weissberg and O’Brien, 2004; Mental Health Commission of Canada, 2012; Trucano, 2016) because it guides SEL programs toward meeting the needs of children, as well as school board needs.

Dinkmeyer et al. (2015) outline the importance of looking at the whole system a child lives in when approaching a child-related problem. They report that, by involving the parents, teachers, and school board representatives in any school-based interventions, the whole system is being addressed, altering the environment in which the child develops—reinforcing research that shows parents who have a positive relationship with their children’s school and are emotionally connected to their children have children who are more likely to have proficient social-emotional skills (Kerns et al., 1996; Clark and Ladd, 2000). Highlighting the importance of having parents’ and schools’ support cultivates social-emotional skills (Kerns et al., 1996; Clark and Ladd, 2000). Finally, by involving the stakeholders such as families and school board members throughout the research process, the research to action gap is decreased, improving the quality of care for the children (Graham et al., 2006).

### DREAM Online-Live Hybrid Adaptation

The purpose of the present study is to adapt DREAM to an online-live hybrid through the KTI engagement of families, teachers, children, and school boards. The live version of the DREAM program was evaluated with a group of students ages 6 to 16, their parents, and teachers, and that study found that the DREAM program was effective in reaching it’s goal of cultivating resilience (Armstrong et al., 2020). Specifically, it enhanced openness to learning and to feelings, hope for the future, self-esteem, and agency over thoughts and behaviors, promoting both internalizing and externalizing mental health (Armstrong et al., 2020). The motivation for the adaptation proposed in the present study came from the results of a KTI evaluation of an earlier edition of the program (Armstrong, 2017). It was recommended that the program should be delivered online to extend program reach but be carried out within a group setting, like a classroom, in order to retain the group-based games and activities— promoting meaningful social engagement (Armstrong, 2017).

Developing an online-live hybrid of DREAM would increase accessibility to the program because of the decreased cost of not having a psychologist facilitate the program, rural school boards where there is a high mental health need would be able to implement it, and the use of technology caters to the lifestyle of the target population. Over 90% of youth use computers and two-thirds of adults do (Ybarra and Eaton, 2005). In addition, many programs involve “train the trainer” models in which extensive training is required to implement the program, or they involve detailed implementation manuals which can impact implementation fidelity, or programs otherwise come to an end when research ends (Lean and Colucci, 2013). The creation of an online-live hybrid will hopefully serve to address these inaccessible gaps of service. Specifically, the online-live hybrid SEL program enters the world in which children are already living and is resource-friendly in respect of staffing and training.

#### Online Programming and Use of Technology

In a systematic analysis of technology use and beliefs about teaching, Tondeur et al. (2017) identified that the relationship between pedagogical beliefs and technology use is bi-directional, meaning that integrating technology into the classroom can change teachers’ beliefs toward a more constructivist approach and constructivist beliefs can lead to increased use of technology. The significance of educators’ beliefs in the implementation of technology supports the KTI framework, which has constructivist roots. They also identified time as a barrier to technology implementation. The authors found that although professional development was a key problem to technology integration, not all teachers responded positively to professional development; Tondeur et al. attributed this to the complexities of pedagogical beliefs and the resistance to change. The authors stated long-term professional development was indeed needed to change pedagogical beliefs. A method of changing teacher’s beliefs involves providing them with helpful tools such as manuals to help explain how the technology should work; however, an overly detailed manual diminishes program fidelity (Lean and Colucci, 2013). Diminished program fidelity means that the program may not yield its intended outcomes if a program is not implemented as intended (Lean and Colucci, 2013). Tondeur et al. (2017) found that a supportive school environment that promotes meaningful technology integration would produce an effective technology adoption method. The authors noted that stakeholders such as school board members, parents, and administration should be included in discussions regarding how technology would be implemented to create meaningful integration. This research demonstrates that there could be resistance in the uptake of an online-live hybrid program due to the teachers’ beliefs about technology, time restrictions, as well as a lack of professional and environmental support. All of these factors should, therefore, be considered when adapting DREAM to an online-live hybrid model. The literature also notes that barriers to implementing technology-based programs include:

• The gap between technologically proficient youth versus the institutional nature of schools that may hinder use in a manner that best fits youth (Olofsson et al., 2015).
• Access to technology (Olofsson et al., 2015).

Research also highlights how different levels of school structure can impact the teachers’ ability to implement a digital technological program and, yet, research suggests that benefits outweigh the deterrents (Perrotta, 2013).

### Technology and Mental Health

In a review of online programs focused on treatment and prevention for anxiety and depression in children and youth, researchers found the benefits included: accessibility, program trustworthiness (implementation fidelity) could be ensured because of the automation, running costs of the programs were lower, ability to monitor program processes and outcomes were easier through online programs, the interactive visual nature of online programs was more appealing for children, and there was a reduced need for train the trainer (Calear and Christensen, 2010). This research supports the need for online programming such as the DREAM live-online hybrid. There is a key difference between the studies cited above and the one being proposed. The latter will be a hybrid program, meaning that parts of the program—i.e., the hands-on activities—will be carried out with the children in the environment that the program is taking place (in regular or virtual classrooms with a group of students). The teaching information and activity instructions will be delivered via video, meaning that the program implementation remains consistent. The research from previous studies, however, still accurately informs this project because the key recommendations regarding online programs are applicable to the DREAM adaptation. Bonk (2009) reports that an education system should reflect the society that it exists within. During the pandemic, our society is struggling with mental health and using technology more than ever, and creating an SEL program that is an online-live hybrid reflects society’s current state.

### Originality and Research Question

There was a great need for a mental health prevention program for children, and the COVID-19 pandemic has heightened this need. The translation of the DREAM program to an online-live hybrid should hopefully fill this need if the program enhances meaning and mental health. Armstrong et al. (2019) found that the original DREAM program was effective and earlier iterations (Armstrong, 2017) found that knowledge users would like to have the program in an online-live hybrid format. The DREAM online-live hybrid will be the first SEL program designed specifically for children with a meaning-mindset component and will aim to address accessibility gaps with its hybrid online-live model that can be used in any classroom or virtual classroom. The translation of this program to a hybrid model will use a KTI framework to ensure that the best scientific standards are being followed and the program maintains reliability, validity, and generalizability. Therefore, the novelty of this research has three components that work together to create this unique project. The first is the spectrum of stakeholders involved, the second is the initiative to create an online-live hybrid SEL program to meet the changing needs of schools and students during the pandemic. The third is the nature of the program, the meaning centered SEL curriculum, which is the first of its kind. To assess the scientific standards using a KTI framework, the research question for this study is:

How can DREAM be adapted to an online-live hybrid so that it optimizes the feasibility, credibility, sustainability, and acceptability of the program using recommendations from key stakeholders?

## Methodology

### Research Design

Using the KTI framework, it was important to the authors that the essence of the stakeholder’s comments was captured in the results. The authors decided that integrating grounded theory and thematic analysis was the best way to systematically clean the varied data sources and keep the meaning being the stakeholder’s comments. The thematic analysis outlined by Braun and Clarke (2006) provided a structured procedure for data analysis, while grounded theory, informed by Holton (2010); Hays and Wood (2011), allowed to keep the qualitative data the meaning of the data intact. Memos were a crucial part of integrating these two processes because they tracked the author’s interpretations while keeping them separate from the data itself.

### Participants

This study took place in a Canadian Metropolitan city with the following demographic characteristics of participants:

#### Families

The mean age of the parent participants was 40 years, with a highly educated population (45% of the population reported having a university certificate or higher) compared to the national average of 28.5% of the population having a university certificate or higher (Statistics Canada and Government of Canada, 2019). The largest ethnicity represented is White. Regarding the research city, the city statistics indicate an average income at 6% higher than the national average (Statistics Canada and Government of Canada, 2019). The city’s employment rate was similar to the national average, with the unemployment rate slightly lower (Statistics Canada and Government of Canada, 2019). In total, there were nine families; every family had one parent and one child who participated. However, only six families came to all three administration sessions, and seven families were present to fill out the questionnaire. If a family missed a session, they were given the materials to review at home. None of the families who participated in this administration had any previous experience with the DREAM program.

#### School Board Staff

Regarding school board participants, their age and ethnicity were not reported. Further details about school board staff are presented below.

#### Retired Elementary School Teachers

Three retired elementary school teachers (two females, one male) were presented with the materials for the program.

#### Mental Health Experts

Although a child psychologist and her team developed this program, two psychologists were consulted throughout the process of program development. One of these psychologists was academic, who further consulted families with lived experience of child mental illness to provide recommendations, and the other was the former executive director of well-known child mental health organizations.

### Data Collection

The present study uses secondary data from two research projects, under the Principle Research of Dr. Armstrong and her Ph.D. student Emmalyne Watt. Dr. Armstrong and Dr. Watt had previous funding for DREAM research projects. Dr. Watt was conducting research to create a Wait-list program for children waiting for mental health resources, using the DREAM program’s principles. Dr. Armstrong was doing research to expand the accessibility of the DREAM program. Due to the In total, there were seven different data sets. See the Ethics Approval in Supplementary Appendix B for the participants that were recruited for Dr. Armstrong’s and Emmalyne Watt’s research used in this study.

#### Data Sets

##### Focus groups with school boards

The first four data sets include French and English school board staff members in both the Public and Catholic domains who live in a Canadian metropolitan city. Each focus group had three school board stakeholders present at each, and the focus group duration was 1 h at each board. These are school board staff who oversee the gifted, mental health, and special education programming in their school boards, including psychologists and learning support teachers. These participants were recruited through contact via phone, email, or in person. The focus group sizes are supported in research, indicating that very small focus groups increase participation and depth of responses (Chioncel et al., 2003; Toner, 2009). Toner (2009) found rich content that emerged in smaller focus groups compared to individual interviews and traditional larger focus groups (Toner, 2009). Data set six is the post-program questionnaires.

##### Focus group with parents and children

The fifth data set includes parents and children invited to participate in a focus group for the DREAM program targeting families on mental health waitlists. The participants were parents and children recruited either through local City therapists, notified about the program through the online platform *Psychology Reading Circle*, or through a local gifted program Facebook page. This focus group had seven parents and six children present, and the focus group duration was 3 h.

##### Survey data from parents’ post-administration

The sixth data set included the seven families who completed the questionnaire and participated in a previous live administration of DREAM. A questionnaire was distributed after completion, with a portion assessing the participants’ recommendations for the program’s online adaptation. The families for this administration of the program were recruited through local therapists, who were notified about the program through the online platform *Psychology Reading Circle*, local gifted program Facebook page, and through the waitlist focus group. In total, there were seven families present for the live administration, which included four gifted children, one child on the autism spectrum, two children with learning disabilities/ADHD, and the rest were non-identified.

##### Field notes and comments from retired teachers and mental health experts

The seventh data set includes feedback received from retired teachers and mental health experts involved in an interactive reviewing process with an outline of the online-live DREAM program. The online-live DREAM program provided to these stakeholders was a version of the program that had been adapted with the results from the first six data sets. The review process took place after the initial results were obtained and the development of the videos and scripts occurred. The data collection for this data set included notes made on a Microsoft Word document.

##### Description of online-live DREAM program

DREAM includes ten units, each with an original song, discussion, and activities associated with the unit topic. In the online-live hybrid version, the song is going to be made into a music video and will provide the content for each unit. The live portion of the unit includes the discussion led by the facilitator, the games, and activities (i.e., crafts). There are suggestions within the units for take-home or in-class activities. These activities can be used as reinforcement in between units if the facilitator does not deem homework appropriate. As a part of the program, an evaluation would be distributed to the students after they completed the program. Below are the topics for each unit. For more details of the units and content, see Supplementary Appendix A.

*Online-live DREAM Outline.*

• Unit 1: Mental Health and Gifted Literacy.
• Unit 2: Emotion Recognition and Social-Emotional Literacy.
• Unit 3: Relaxation.
• Unit 4: More Calm Down Activities: Worry Time, Imagery, Humor.
• Unit 5: Avoidance and Obsessive Behaviors.
• Unit 6: Enjoyable Distraction.
• Unit 7: Meaningful Living.
• Unit 8: Connection Between Thoughts and Feelings.
• Unit 9: Choosing to Think Differently.
• Unit 10: “Act as If,” Helpful Problem-Solving, and Putting it All Together.

#### Satisfaction Survey

In the satisfaction survey seen in Supplementary Appendix D, there are qualitative and quantitative questions. The researchers decided to focus on the survey’s qualitative data to translate the program to an online-live hybrid. The purpose of this was to produce succinct results as there were many sources of qualitative data. The satisfaction survey it’s self has been created using the principles derived from Armstrong’s (2009) which established the effectiveness of creating an evaluation survey based off the principles of Patton’s (1984) utilization-focus framework. This survey demonstrated face validity as seen in Armstrong et al. (2019), when it was used to establish the satisfaction of participants in the original DREAM program.

#### Data Collection Procedure

No names were included in the focus groups’ transcription or recorded on DREAM participants’ questionnaires to ensure confidentiality. The questions asked in the focus groups and questionnaire pertained to the acceptability, credibility, sustainability, and feasibility of the proposed online-live implementation and more open-ended questions regarding suggestions for online delivery to facilitate knowledge translation. See Supplementary Appendix C for the DREAM focus group guide for school board staff, Supplementary Appendix D for the satisfaction survey items, and Supplementary Appendix E for the relevant DREAM Waitlist focus group questions with parents and children.

#### Consent

The Family Waitlist focus group provided verbal and written consent (see attached consent form in Supplementary Appendix F). The School Board members’, retired teachers, and mental health expert focus groups included both passive and verbal consent. The passive consent was received by the school board members’ response to the email, inviting them to participate. Verbal consent was received at the beginning of the focus group (see script with verbal consent in Supplementary Appendix G). The live administration of the program with families, which included the waitlist addition, received consent within the pre-administration survey (outlined in the form attached in Supplementary Appendix H). The physical copies of the questionnaires are kept in a locked office, in a locked filing cabinet. The data extracted from the paper questionnaires were coded, and the transcript from the focus groups was encrypted and saved on a USB which is kept in a locked filing cabinet in the locked office.

### Data Analysis

#### Theoretical Framework

This is a qualitative study using grounded theory and thematic analysis (Hays and Wood, 2011). Thematic analysis is a deductive process, which involves exploring common patterns and themes that arise from the data (Vaismoradi et al., 2013). A theme is defined as different parts of the data are grouped in a coherent way that uncovers new information about the research question (Vaismoradi et al., 2013). In this case, the main categories were accessibility, feasibility, credibility, and sustainability, aligning with the KTI framework. Within the literature, there is confusion surrounding the difference between content analysis and thematic analysis. Authors Vaismoradi et al. (2013) explain that content analysis can only explore manifest or latent content, whereas thematic analysis is a fluid integration of both. The authors expand on this model, stating that manifest content is patterns that are obvious in the data, whereas latent content depicts meaning derived from the patterns in the data. Thematic analysis was chosen for this project because of the ability to integrate the meaning of the content, along with the analysis of the content.

Grounded theory has roots in symbolic interactionism and is a deductive process, which means that when an interpretation of the data occurs, the philosophical and societal bias of the researcher is embedded into the results (Ralph et al., 2015). Due to this interaction between the research and the data, different ways to address this have been developed. Berger and Kellner (1981) suggest that whoever is analyzing the qualitative data should be aware of their reactions and biases to the data and attempt to put these aside. Other researchers suggest that it is this interaction that creates socially relevant results within the grounded theory (Turner, 1981; Stern, 1994). Cutcliffe (2000) argued for the integration of these ideas, encouraging the awareness of values, previous knowledge, and ideas to interact with the data, producing a creative approach. The trustworthiness of the results is validated through the grounded theory methodology, which happens when emerging ideas are confirmed in parallel data sources (Cutcliffe, 2000). The grounded theory approach is complimentary to thematic analysis because both incorporate the context and environment in which the data exists. This integration is supported by Wilson and Hutchinson (1991); Stern (1994), Cutcliffe (2000) because the combination of methodologies allows for a wider and deeper exploration of the data. An outline of how the thematic analysis and grounded theory were combined for the analysis can be found in Table 1.

**TABLE 1:** Description of data analysis procedure.

Stage	Thematic Analysis (Braun and Clarke, 2006)	Grounded Theory (Holton, 2010)
(1)	Become familiar with the data.	Create initial memo’s that describe initial reactions to the data.
(2)	Begin open coding	Substantive coding occurs, which is where the data is broken apart. This process includes open coding. Memos are created throughout this process. This step is repeated multiple times.
(3)	Search for main themes emerging from the open codes.	A core variable is identified and memos are created.
(4)	Start to compare and contrast themes to other emerging themes.	Selective coding begins after the core variable is chosen. Selective coding compares the incidents and the properties of codes.
(5)	Define themes, ensure that themes are coherent, distinct from each other, a good fit and identify any sub themes,	At this point, theoretical saturation should have occurred. Memos are analyzed and used to tie together existing codes at a higher conceptual level.
(6)	Extract quotes that represent the themes, report on the existing themes and how they relate.	Presentation of the core variable and other codes, along with the researcher’s conceptualizations.

#### Coding Procedure

With the integration of different coding methodologies, precision can be compromised (Wilson and Hutchinson, 1991; Stern, 1994; Cutcliffe, 2000). To avoid this, the author carefully followed the integrated procedures outlined in Table 1. and included multiple informants analyzing the data. The data analysis followed the thematic analysis outlined by Braun and Clarke (2006) in conjunction with grounded theory analysis informed by Holton (2010); Hays and Wood (2011). Data analysis was conducted by three researchers to ensure validity through triangulation. As the main part of grounded theory is the interpretation of the data, the best standards of practice are for the socio-cultural context of the researchers analyzing the data to be described. The lead researcher is a Counseling Masters Graduate, identifies as female, is White, and lives in a Canadian metropolitan city. The second researcher to analyze the data was a professor and clinical psychologist at a university in a Canadian metropolitan city, who identifies as female and is White. The third researcher holds an undergraduate in psychology, identifies as female, and is White. There were two female identifying translators who provided cross-informant translations of the French data to make sure the translations matched. A further translator was male identifying, giving the interpretation of the data a more gender-balanced perspective.

Second-tier triangulation is an additional assurance on validity, which involves multiple methods of collecting the qualitative data (Finfgeld-Connett, 2010), which is demonstrated in this project using a questionnaire and focus groups. These steps, along with the assessment of the interrater agreement aimed to address and minimize bias from the researchers, support the validity and reliability of the analyses. An inter-rater agreement of 75% was considered acceptable (Statistics How To, 2016), and the interrater agreement that was achieved was 95%, with the 5% of disagreement existing due to choice of words that were considered synonyms. NVivo 12 software was used to facilitate data analysis. Corresponding to the NVivo analysis, the researcher, research assistant, and volunteer also followed this procedure of analysis manually.

## Results

The information provided in this section was produced from a Methods Diary and memos that were kept by the author throughout the data analysis. As per the KTI lens, this project used the main four categories of Feasibility, Credibility, Sustainability, and Acceptability, and general themes emerged from the data that fit these categories. The definitions of these categories evolved to better suit the emerging data throughout the analysis process. The evolution of the definitions can be seen in the *definition table*, Table 2. The data analysis used a combination of thematic and grounded analysis; three sub-categories emerged within each main category (Feasibility, Acceptability, Credibility, and Sustainability); the sub-categories that emerged included: concerns, recommendations, and validation. Paraphrased field notes and direct quotes were used to illustrate these categories and sub-categories.

**TABLE 2:** Definition table.

	1st	2nd	3rd
Acceptability	Uses the literature review and stakeholder opinions	Shows consistency between literature review and previous stakeholder opinions	Willingness to use the program (it’s appealing and meets stakeholders needs)
Feasibility	Able to do program from a time and resource standpoint	Could do the program again	Perceived ability to use the program
Sustainability	Little external support	Would like to do the program again	Likelihood to use the program, meets long term goals
Credibility	The program does what its supposed to do	A goal of the program was achieved or could be	A goal was achieved, or could be

### Data Analysis Strategy

This is a narrative outline of how the author conducted the data analysis and coding that parallel’s the methods outlined in Table 1:

1. The author familiarized herself with the data, reading and re-reading the transcripts and surveys.
2. Once each document had been read and preliminary memos were made, the data was uploaded to NVivo.
3. Using the KTI lens, each comment or grouping of comments within the data was categorized according to the KTI pillars of Feasibility, Credibility, Sustainability, and Acceptability.
  a. While doing so, making memos of any emerging sub-categories.
4. Further definition of the principles followed and decision about which comments may belong in each main theme.
  a. Established relationships between categories and the differences among them.
i. Differentiating between codes:
1. Categories that emerged in feasibility hold similarities to categories that emerged in credibility. The difference in emphasis is that credibility focuses on long-term goals.
2. There is also a significant cross-over between acceptability and sustainability. The author differentiated these two by looking at acceptability as the stakeholder noting the program’s face validity and sustainability, integrating this face validity with the goals of the program, creating sustained learning. Sustainability is a different form of credibility because credibility deals only with the goals of the program.
5. Label categories concerns, recommendations, and validation and categorize the data accordingly.
  a. Within Microsoft Word, the author noticed which specific comments emerged in multiple categories and re-categorized them according to the differentiating definitions listed above.
  b. Establish relationships between the categories and the sub-categories and differentiate specific comments accordingly.

• The author noticed that comments regarding linking the program to the curriculum appeared across feasibility, credibility, sustainability, and acceptability. The author reviewed the main category’s core values and allocated the theme of curriculum comments into Feasibility and Credibility. Curriculum links would make the program easier to implement, increasing feasibility while also contributing to the reinforcement of the program’s values by connecting the program’s material to material the children are learning throughout the year.

### Reporting the Results

The main categories are feasibility, credibility, acceptability, and sustainability. The sub-categories include concerns, recommendations, and validation. There are themes that repeat in different categories and sub-categories; this is because, in data that emerged, the labels for themes were appropriate in different categories. However, the value of the data is what dictates what categories the themes were assigned to. The value of the data is the essence of the quote or field note and was established by the author reflecting on the core values of the main categories. As depicted above in the data analysis strategy, there are differentiating values within each main category. See below the main values that led to the differentiating factors:

– Feasibility: Factors that contribute to the perceived ease in using and implementing the program.
– Credibility: Factors that contribute to achieving or reinforcing the short-term goals of the program.
– Acceptability: Factors that contribute to the face validity of the program.
– Sustainability: A combination of achieving face validity and long-term goals.

Therefore, while reviewing the themes in each sub-category and category, consider that while the themes may repeat, the value of the data varies with the category the theme belongs to.

### Feasibility

Within the three sub-categories of feasibility (concerns, recommendations, and validation), a variety of themes emerged. Below are the definitions of each of the themes. Find the quotes and field notes that support these themes in Table 3.

**TABLE 3:** Feasibility qualitative results.

Feasibility Categories	Quotes and Field Notes Depicting Themes
Concerns	Implementation
	– “Because when we launch something for teachers. to say, ’well, here’s what you can do’. we have to remember that the teacher is already overspent.” School board 3 – [if implemented with only higher needs children, such as five gifted children in a class] “Supposing I have a class of 25, and I choose 5 to whom I want to offer it. What do I do with the other 20 students During that time?” School board 3 – School board 2 discussed having the program be translated to French so their | French-Immersion gifted classes can participate in the program as well Technology – “[…] it would be important to assure that it would be possible to view them [the videos]. Not every class has a screen or a television.” School board 3 – “There are schools that still don’t even have useable internet access.” School board 3 Duration – [if units are grouped together rather than administered one at a time] “Smaller doses, 2 h was a lot for my kids.” Post-Administration survey – [if units are grouped together rather than administered one at a time] “I do not think an hour and a half, we do not have the time in an hour and a half and then we will not have the concentration and engagement of the student for an hour and a half.” School board 4
Recommendations	Curriculum
	– School board 2 made direct recommendations regarding which units could relate to which exiting school curriculum. – “Also make it appropriate for the amount of parental support for the age group. Less with older kids and more with younger kids.” Waitlist focus group Facilitator – “That does not mean it’s going to be the teachers. It can be a resource teacher. As I said it can be an educator.” School board 4 – “Our special education counselors are there every week or two weeks. So, if they give themselves ten weeks or six weeks, depending on the modules, with a certain group there are several ways that it can be delivered.” School board 4 Duration – “5-10 mins- any longer and may become distracted with other things/no longer engaged.” Post-Administration group – For the take-home activities, parents indicated wanting to know how long they would take. “If there’s a program please do indicate how long it will take.” Waitlist focus group
Validation	Curriculum
	– “I see a lot of cohesion and alignment with our curriculum and existing programs.” School board 1 – “There are several things in there that I see in the health curriculum at the level of physical education and health.” School board 4 Technology – “[…] all our schools have Chromebooks.” School board 4 – “The bandwidth has been expanded in schools” School board 4

– Implementation: Describes factors of incorporating the online-live version of the DREAM program into schools.
– Technology: Considerations surrounding technological requirements.
– Duration: Considerations about the program’s length.
– Curriculum: Factors considering the incorporating of the program into the existing curriculum.
– Facilitator: Considerations of who would be facilitating the program.

### Credibility

Within the three sub-categories of credibility, a variety of themes emerged. Find the quotes and field notes that support these themes in Table 4.

**TABLE 4:** Credibility qualitative results.

Credibility Categories	Quotes and Field Notes Depicting Themes
Concerns	Online-Live Viability
	– Two out of seven parents who participated in the live administration expressed concern about achieving the goals of the program through an online-live hybrid with one stating: “I think in-person with groups is the most effective delivery program.” Implementation – School board 2 members indicated that a barrier is whether the staff can imbed the program into the current curriculum. Unit Specifics – As the avoidance activity in one of the units was going to be presented in a video, a new group activity had to be created for the unit. School board 2 reported that the proposed activity about avoidance would not be a good fit, so they suggested using a read-aloud story instead. School board 1 suggested that using bubbles to teach relaxing breathing would not be appropriate, so they proposed using a different activity called five-finger breathing. All other activities were perceived as appropriate
Recommendation	Implementation
	– School board 1 reported that, during EQAO testing year (Grade 3), when anxiety is higher in students, this would be a good fit group for the program – School board 1 suggested that the program fit well with Phys Ed (socio-emotional learning curriculum), Language Arts, and Family Life (relationships/feelings) classes – School board 2 corroborated the ability to link the DREAM program – Gifted Edition to Health, Physical Education and Literary courses
Validation	Implementation
	– School board 1 members reported that when schools can see how deep learning can be supported through the well-embedded content, the buy-in is high. They then agreed that DREAM – Gifted Edition could be effectively implemented and achieve the goals of the program – “What we already do responds to these objectives. So, if this program can add to, or give new avenues to the workers, it will for sure cover these objectives.” School board 3 – “There are several things in there that I see in the health curriculum at the level of physical education and health.” School board 4 Goals – Five out of seven parents who attended the live administration agreed that developing online videos with printable discussion topics and activities could help achieve these goals, to enhance mental health and meaning. Regarding the other two parents, one parent indicated a preference for in-person administration and the other participants stated, “I’m not sure.” See Table 3 for the parents’ specific comments – Parents who attended the live administration also reported that the goals of the program were reached. Indicating that they were given “Useful techniques” and that the program “Made me more patient/Kids used it too.”

– Online-Live Viability: The perceived ability and impact of the program being an online-live hybrid.
– Implementation: Describes factors of incorporating the online-live version of the DREAM program into schools.
– Unit Specifics: Data that directly comments on parts of specific units.
– Goals: Quotes and field note that directly comment on the goals of the program.

### Sustainability

Within the three sub-categories of sustainability, a variety of themes emerged. Find the quotes and field notes that support these themes in Table 5.

**TABLE 5:** Sustainability qualitative results.

Sustainability Categories	Quotes and Field Notes Depicting Themes
Concerns	Overview
	– “Based on this brief overview, I have difficulty in saying whether or not it is maintainable or not.” School board 3 – “We must try it. You really have to try to see how long it takes, who can give it, accessibility to the tools too. It is really after having tried it we will be able to say whether it is.” School board 4
Recommendations	Memory Aid
	– School board 2 members suggested that the children have a take-home manual for themselves, like booklets with key messages and have a teacher wrap up at the end of the program – Parents who attended the live administration suggested “Maybe more memory aids/mnemonic aids to keep all the tools in mind. When problems arise.” – School board 1 discussed having reinforcement activities between units. Specifically, all the pieces of the current program that are homework could be used in class as reinforcement exercises between weekly/biweekly main lessons Unit Specific – “More exercises to help children identify when they are ’starting’ to feel these big feelings.” – “Not sure how you could do this but more identifiers, so the child knows they are starting to feel these big feelings. So they are aware they are going to be able to ’control’ or better manage their feelings (in a safer way).” Movement and Engagement – “It must move!” School board 4 – “Games, play games again” Parent from live administration
Validation	Memory Aid
	– [regarding the take-home booklet that reminds families of skills learned] “Allows me to refer back to the session when dealing with problems.” Movement and Engagement – “I liked the interactive games, it showed us how we could extend this at home” Parent from live administration – “The game ideas, engages the children which is great!” Parent from live administration

– Overview: Quotes and field notes that pertain to the overall program and not one specific factor.
– Memory Aid: Data that mentions using various memory aids.
– Unit Specifics: Data that directly comments on parts of specific units.
– Movement and Engagement: Quotes and field notes that discuss physical activity and engagement.

### Acceptability

Within the three sub-categories of acceptability, a variety of themes emerged. Find the quotes and field notes that support these themes in Table 6.

**TABLE 6:** Sustainability qualitative results.

Acceptability Categories	Quotes and Field Notes Depicting Themes
Concerns	Online-Live Viability – In the survey researchers asked the parents who attended the live administration if they would like this program if it were taught through videos on the computer, but they still got to do the other activities, 4 out of 7 responded with concerns: “Not as much, I’m old school. Face to face is more engaging for me. But I’m sure video could be made to work.” “No- I enjoy a hands-on approach and the opportunity to ask questions if needed (instant).” “No- I wouldn’t do it online. In person is more effective.” “Not as much.” Language – After participating in the live administration, one parent commented on the appropriateness of the language used: “Some of the language was a little tricky (difficult to understand) for my child to understand.” Duration – School board 2 discussed their concerns about how much time the program will take as a possible impediment to program utilization
Recommendations	Technology
	– School board 2 indicated that the use of the Google educational tool Pear Deck would be relevant for the target population (Google, 2019). As per the music videos, School board 2 noted that having visually appealing lyrics in the video with an icon pointing to the lyrics would be beneficial in order to allow for a potential sing-along – School board 4 added that having diverse children in the video is imperative: “I think it’s important that it varies as much as possible and that we have a representation of cultural diversity so that it fits well with the reality of the school board.” – School board 2 mentioned making sure the web interface simple to use – School board 4 stated, “But it must be easy to access a bit like an application that we see on a phone or an IPad.” Unit Specific – School board 2 went through almost all of the units and indicated how acceptability can be improved. The list below summarizes some of the key recommendations: – In general, use, use terms parents/guardians when referring to the caretaker of the child – Unit 3- Bubbles would be too messy, instead of practice deep breathing by counting to five using fingers, or other deep breathing exercises already being practiced in our board – Unit 5- After watching the video modeling behavior about petting dogs, there should be no personal discussion about fears. Also, make sure to mention that they have to ask before petting a dog – Unit 6- After the song about distracted verse helpful thinking there should be a discussion about the difference between the two thinking styles – Unit 7- Simplify the instructions – Unit 8- The crown activity should be done live Facilitator – School board 2 suggested having a clear and structured guideline for the discussion and activities which include: prompting questions, guiding questions, and examples of responses, guide ideas about how to respond and make sure teachers are supported in ideas of responses. They also noted that a brief user-friendly manual would enhance teacher and school board interest in the program uptake – “Because the group of workers exists already. These people are always searching for modules or content to address certain challenges.” School board 3 Time – “Has to fit within class time.” School board 2
Validation	Content
	– School board 1 reported that the group activities were a good fit – School board 2 stated “Overall video provides consistency” and “overall sounds like fun!” – School board 4 stated that the DREAM program – Gifted Edition addresses more topics than existing programs, “There are points within this, modules in this, that go further than modules that I’ve seen before.” – Parents addressed the age-appropriateness of the program, “It was good because it wasn’t just for older kids or little kids.” Technology – Of the parents who participated in the live administration, 3 out of 7 indicated that they would like this program if it were taught through videos on the computer and they were still able to do the other activities live – Parents from the Waitlist focus group reported on the inevitability of technology being integrated into their children’s lives stating, “Because it is becoming more of their lives it’s important to incorporate it.” And indicating “I find my [child’s] teachers right now are very much incorporating using video, iPad, online things for teaching and even for adult learning were using simulation for learning. We might as well keep embracing it.” Games and Creative Activities – Comments from the parents and children who participated in the live administration: “The game ideas- engages the children which is great!” “I liked the interactive games.” “I liked the art stuff and the active stuff.” “I enjoyed the freedom of movement while we sang.” “The songs and the different exercises/games that you can do with your child.”

– Online-Live Viability: The perceived ability and impact of the program being an online-live hybrid.
– Language: Comments on the level of language used in the program.
– Duration: Considerations about the program’s length.
– Technology: Considerations surrounding technological requirements.
– Facilitator: Considerations of who would be facilitating the program.

Content: Data that pertains to the content of the overall program.

– Games and Creative Activities: Quotes and field notes about games and creative activities used in the program.

### Teachers and Mental Health Experts

Following the interpretation and application of the above qualitative data, a proposed live-hybrid model was constructed in further collaboration with school board staff. Additionally, video scripts were written by a professional media production studio using the existing DREAM administration materials and feedback from the above stakeholders as the teaching template. Further materials (e.g., read-aloud stories written by a child and a psychologist) were developed to fit the adapted program suggestions from the stakeholders. Three retired elementary school teachers and two mental health experts were then consulted on an ongoing basis to review the video scripts, materials, as well as the resulting video episodes that were produced^2^. See Table 7 for comments made by the retired teachers and mental health experts.

**TABLE 7:** Retired teachers and mental health professionals qualitative data.

Topics	Field notes and comments from stakeholders
Episodes	– Any group of primary, junior classes would easily relate to those situations. Lots of room for talking points as well. They did a really good job
Read aloud materials created	– The unit is nicely integrated with the story and poem. Great stories - easily relate to the aim and the objectives. The student will certainly be able to relate to the read-aloud story and poem – The Worry Wind is an exceptional poem. It really covers the feelings of worry/anxiety so well, and I think students of a variety of ages/grades would connect to it. I would say especially grades 4-6 and perhaps even grade 3. It should prompt many discussions around anxiety – The story is one with which students of the same age group would identify. I would preface that it is written by a Grade five student. I would even get students to take turns reading it. It all wraps up to a positive ending, and I like the messages throughout. These will be good ways to go over the choices (Bad and good) one could make in the same situation. (Telling a parent, having a bystander stand up to the bully, being honest and being yourself) it may even prompt some writing about similar situations. (Combining Health class with language!)
Brief program manual for educators	– Quite interesting. In fact, follows exactly what the guides for most of our course outlines would be. Definitely would be able to follow this course with your format. Good to present the purpose for each unit
Scripts	– I could see these being really helpful for younger grades in elementary school. The vocabulary and activities are at an appropriate level for that age group. I also think the fact that there are kids their age in the videos, having the discussions and even creating some of the presentations will be appealing. Honestly, teachers will lap this program up! Mental health issues are at the forefront in many classrooms. So many kids have so much going on and those who aren’t coping will likely be the ones who suffer most. But my experience is that the rest of the class suffers as well. I think it is important to have these discussions not only to get it out there in the open, so that kids know they are not alone, but to help everyone cope and to be more empathetic if others around them are hurting

## Discussion

To answer the research question of how to translate DREAM to an online-live hybrid best while optimizing the principles of the KTI framework, the authors integrated concerns noted by the school board members and parents with their own recommendations, as well as recommendations made within the literature. Within each section of feasibility, credibility, sustainability, and acceptability, stakeholders’ validation was summarized, indicating what stakeholders liked and, therefore, what should be continued throughout further program development. The author noticed a few characteristic differences between the different stakeholders’ types of comments while analyzing the results. The families in the focus group and who attended the live-administration mainly commented on the nature of participating in the program. For example, they commented on the duration of the program and the content of the program. Whereas the mental health experts, retired teachers, and school board members discussed program duration and content, how the program implementation, and more functional aspects of program translation. These differences are important because they speak to the value of having different types of stakeholders evaluate program translation. Overall, support for the online-live hybrid was observed, and buy-in from four large school boards was generated. All the school boards requested the program within their classrooms after participating in the focus groups. Through collaboration with key stakeholders, suggestions were provided in the areas of feasibility, credibility, sustainability, and acceptability.

### Feasibility

To increase the perceived ease in implementing an online-live program, one school board indicated that the program could be run by resource teachers or special education staff if conducted with select groups of students (e.g., gifted pull-out groups), in addition to carrying out the program in regular classrooms or behavioral classrooms. School board staff who could see clear curriculum links perceived the ease of implementation within classrooms by teachers. By contrast, school board staff who were unaware of curriculum links as they were less familiar with the program had concerns regarding implementation by teachers who already have many responsibilities. It leads that familiarity with the program increases perceived ease of implementation.

Generally, it was recommended that the whole class participate in the program, as school boards perceived that the skills taught were relevant for all students. Collectively, even though DREAM was originally designed for intellectually gifted students, whose unique characteristics put them at increased risk for mental health concerns (Armstrong et al., 2020), all the school boards wanted to apply for the program beyond their gifted populations. Once the pandemic hit, it made the structure of the program even more relevant. Thus, the focus of this article was on children in general, rather than gifted children, and the online-live hybrid was adapted for all students. After *school board 2* mentioned wanting the program for their French-immersion classes, the researchers included the French school boards as stakeholders in focus groups. This led to the translation of all the units, the songs, and the music videos. This increased the program’s feasibility by decreasing language as a barrier.

Access to technology was flagged as a concern of the usability of the program, and this concern varied between school boards. Most of the school boards reported that their schools have a great deal of access to technology, but one school board requested that the program be offered on a USB stick for schools with less technological access. The research did indicate that access could be a barrier. Access to resources is a function of funding that varies between school boards and could explain the differing concerns between school boards (Olofsson et al., 2015; Riel et al., 2016; van Lieshout et al., 2017). DREAM is designed to be low-cost regarding in-class activities, only requiring materials that would be readily found in the classroom, such as paper, markers, and scissors. It could be useful when implementing the program to ask schools specifically what they would require for the online portion to be easily adaptable to their system and see how this can be accommodated.

For the school boards where Internet technology is readily available, facilitation of the program is reportedly simple: Staff clicks on the weblinks for the videos for each program unit. For the schools where the internet is limited, the program’s online portion would be provided on a USB stick. For the schools where televisions are limited, it is suggested that schools should be aware of the number of sessions needed to complete the program in advance in order to facilitate renting a television, as school board stakeholders suggested. This would look different for each school as they each have different needs.

Along with some concerns regarding access to technology, some school board members discussed how the technology requirements would be easy to implement and indicated that they saw ways that the program related to the existing curriculum. These factors demonstrate how the online-live hybrid is viable with the existing resources in some cases.

### Credibility

Just as Tondeur et al. (2017) outlined how the beliefs that teachers hold can impact the implementation of technology in education systems, it can be inferred that parents’ beliefs surrounding technology can impact how they implement technology within their home. A minority of parents reported skepticism with the online version reporting that they prefer face-to-face programming. However, this program is designed to be administered in a school setting, and the online-live hybrid has the opportunity to balance the concerns of the families with the practical issues of a busy curriculum. Many parents discussed the importance of technology today, and with any change in the zeitgeist, there are going to be those who resist the change. Out of the three Saturdays, the program was administered, only six out of nine families were able to attend all three sessions. The families who were not able to attend were presented with the units they missed for at-home completion. The authors recognize the hesitance of incorporating more technology and emphasize the hybrid nature of the program, with the facilitator providing the face-to-face activities and discussions to achieve the meaningful social engagement goals of the program. In a COVID-19 setting, this face-to-face portion of the program would be incorporated into the virtual classrooms that school boards are using.

Research indicated that, for technology and the goals it wishes to achieve, in this case, SEL with a meaning-mindset component, the whole system needs to be involved (Durlak et al., 2011; Jones and Bouffard, 2012; Dinkmeyer et al., 2015; Tondeur et al., 2017). This looks like a systemic approach to implementation that reinforces the program’s goals at all levels of the structure. When this is done properly, the goals of the program are not only reinforced with each session but throughout the child’s day at school. Regarding the current proposed online-live program, although the program units would be implemented and reinforced at school, parents also would receive information regarding what children have learned in order to further reinforce these skills at home.

The program demonstrates high credibility with the stakeholders, suggesting that the program meets their needs. One parent commented that the program “Made me more patient […].” These validations are echoed throughout the acceptability section, where school board 4 commented on how the program “[…] go[es] further than modules that I’ve seen before.” This overlap in credibility and acceptability illustrates how the goals of the program inherently fit with the needs of the stakeholders. The schoolboard’s perceived program acceptability is reinforced by the parents noting that the goals of the program were achieved during the live administration. This validation cycle affirms previous studies that have established the program’s efficacy, as well as supports the mission of the present study to translate the program to an online-live hybrid. Parents also reported wanting to see the program widely implemented, which aligns with stakeholders’ suggestions in previous research (Armstrong, 2017).

### Sustainability

There is ambiguity surrounding stakeholder’s ability to comment on the sustainability of the program because they have not seen exactly how the online-live hybrid works in their schools. Stakeholders did formulate suggestions to ensure sustainability, which includes: incorporating memory aids such as booklets with key takeaways between unit activities and mnemonics, exercises that help identify the start of feelings, adapting the avoidance game to a read-aloud story, as well as ensure there are many games and a great deal of movement. It is the author’s and stakeholder’s view that if the program is implemented with the recommendations made by key stakeholders, the program will inherently be sustainable. If once the program has been run, it is found to not be sustainable, there are implementation changes that the school can make so that it addresses any potential issues without affecting implementation fidelity (e.g., who implements the program, who receives the program, how often the program is implemented). Parents who completed the live version of the program directly stated that they have regularly referred to the skills they learned during the program suggesting that the program likely has the ability to sustain the intended goals.

### Acceptability

Some parent stakeholders indicated that face-to-face programming is important to them with respect to their willingness to use the program. There was no research exploring the parent’s beliefs about using technology in education. The Tondeur et al. (2017) study demonstrated that after teachers engaged with technology, they expressed more positive opinions toward technology. If the same logic is used with parents, then with engagement in the online-live hybrid using the constructivist approach of engaging parent stakeholders to inform the program development, parents who are skeptical might express more positive opinions as well.

School board stakeholders made technology-specific recommendations that would increase the program’s usability. These recommendations include display lyrics in the music videos with an icon above the lyric indicating the tempo, online platforms should be easy to access like applications, using Pear Deck if it already exists within the school board (Google, 2019), and unit specific recommendations. The unit-specific recommendations were simple and concise, making them appear to be easy to integrate. Stakeholders also recommended there should be diversity among the children in the music videos that each unit of the program uses to reinforce information learned. This aligns with Bonk’s (2009) evaluation, which notes that in the twenty-first century, the technology used in schools should be representative and mirror users’ lived experiences.

Research on how to support facilitators in implementing an online-live program is varied. Tondeur et al. (2017) reported that although some teachers’ beliefs became more favorable toward technology after professional development, not all teachers’ opinions changed. This demonstrates how hard it can be to adapt a person’s beliefs and that professional development does not always work. The program’s plan to assist facilitators is through a manual that will be provided detailing each activity, as well as brief video-based demonstrations of exercises for teachers. From stakeholder comments, it is clear that supporting the facilitator is important and, with this, a fine balance should be struck, as research also shows that an overly detailed manual diminishes implementation fidelity (Lean and Colucci, 2013).

School board stakeholders indicated specific areas they believed a manual could best support their teachers, which were consistent with the research (Garinger, 2010; van Lieshout et al., 2017); those included: prompting questions, curriculum links, examples of responses and ideas about how to respond. It is the author’s recommendation to include each of these areas in the manual.

After either participating in the live administration or after hearing a detailed description of the program, stakeholders reported that the program was appealing to them for reasons including interesting and age-appropriate content, depth of learning, use of technology is consistent with current school trends, as well as fun and engaging crafts, games, and activities. The acceptability of the program is important because when a program appears to be helpful, engaging, and easy to use, the knowledge users are more likely to use and benefit from the content of the program (Mental Health Commission of Canada, 2012).

### Limitations

The conclusions of this study have many important applications, but, as with any study, it has limitations. The demographics of the stakeholders are representative of the city where the study took place. However, they are not representative of the whole province or country. With the city population being majority White and the socioeconomic status being higher than the national average, the results of this study would not capture the lived experiences of minorities in Canada and those with a lower socioeconomic status. Therefore, future research should include a more diverse set of stakeholders. There is also a lack of representation for rural stakeholders, both parents and school board members. It is possible that, although some recommendations might be similar, there would be different considerations for rural versus urban environments. This concern is relevant as rural children may benefit the most from resilience programming, given higher mental health concerns in these areas, as well as less access to mental health services (Armstrong, 2011).

While transcribing and analyzing the data, it became clear that there was some confusion around the structure of the program. This led to some recommendations that were not relevant. This lack of clarity could be due to the fact that one focus group involved a change in participants shortly before the group happened (e.g., mental health leads at a school board were called away, so had others sit in their place). This meant that the program had to be described to the participants in detail, but the participants were making comments before they fully knew about the program. For future stakeholder engagement, it is recommended that designated time be assigned to explaining and detailing the structure of the program. This will hopefully lead to more relevant recommendations and more clarity for the stakeholders.

A similar confusion happened with the parent stakeholders regarding the perception of program formatting. Due to time restrictions and stakeholder availability, the live administration was conducted in 2-h sessions over three weekends, with several units grouped together at a time. While making recommendations, the stakeholders reported that the allotted time was too long. This comment was applied to the results. However, it would have been beneficial if it was made clear to the stakeholders that the format they experienced would not necessarily be the same as it would be administered. In fact, as an online-live hybrid program, schools could choose to deliver a single unit each week, with the reinforcement activities in between, giving time also to apply the related Ministry of Education curriculum.

A further limitation includes the over-representation of females throughout the research process. In the translation of French to English focus group transcripts, transcription of the focus groups, and analysis of the data, all but one of the researchers included were female. Due to the subjective nature of language translation and grounded theory analysis, this research could hold a female bias.

### Future Directions

The next step for this project is the ongoing implementation of the recommendations made in this paper while allowing some flexibility for school board specificities without compromising implementation fidelity. As seen with the comments made by the seventh data set, the retired teachers and mental health professionals, stakeholder consultation is a standard. This is important since the demographic used in this study lacked representation for diverse and rural communities and, therefore, consultation with the incoming setting is necessary. To date, one of the current research assistants and translators is an expert in children’s media, having written scripts and music lyrics for both French and English children’s television programming.

It would be interesting to research how this program fits with minority gifted children to see if the program’s design allows for enough facilitator support and flexibility to cater to the children’s individual needs.

Another interesting research follow-up would be to see the longitudinal benefits of participating in this program. A future planned randomized control trial (RCT) to assess the DREAM Edition program online-live hybrid, in comparison to school programming, as usual, could provide evidence in support of the program or indicate more consultation needs to take place. Within this future RCT, controlling for gender and socioeconomic status could give important insights that may inform further development of the program.

The online-live recommendations can be used to further develop other versions of the program as well, such as a version for families on mental health waitlists, as well as other SEL programs. The recommendations above summarize important considerations stakeholders have while approaching an online-live transition. Therefore, this research can be used to aid other programs looking to transition to or create an online-live hybrid program.

## Conclusion

This study highlights the importance of tuning in to children’s needs and listening to those who support them. Stakeholders offered many valuable concerns, recommendations, and validations that will help shape this iteration of the DREAM program and future additional program versions. This research adds to the existing body of SEL programming literature by showing the value of consulting with a diverse group of stakeholders, the benefits of an online-live hybrid program in this pandemic context, and meaning making as a foundation social-emotional education. Overall, to extend program reach, the present study supports developing an online-live hybrid model for the existing DREAM program, which was previously found to promote internalizing and externalizing mental health (Armstrong et al., 2020). Further, this research supports program implementation for gifted students and all students, particularly during this pandemic time period, where most parents are reporting some mental health symptoms in their children (Fegert et al., 2020). By engaging children and their peers in this resiliency program and making sure the program fits their needs, the developmental challenges associated with mental illness risk may be lowered. Ultimately, it is hoped that this program’s widespread implementation may improve the quality of life for children and their caregivers and lessen the economic burden on family systems and society, who were already experiencing mental health concerns that are now exacerbated by the COVID-19 pandemic.

## Data Availability Statement

The data analyzed in this study is subject to the following licenses/restrictions: Although no names or institutions were recorded, to protect the identity of the participants, only excerpts of the qualitative data was included in the manuscript. Requests to access these datasets should be directed to JP, jparr088@gmail.com.

## Ethics Statement

The studies involving human participants were reviewed and approved by the Research Ethics Board, Saint Paul University. Written informed consent to participate in this study was provided by the participants’ legal guardian/next of kin.

## Author Contributions

JP conducted the literature review, developed and executed the methods, co-facilitated focus groups and the live administration, and produced the results and discussion. LA created the research study, identified the need, built the research team, and supervised the research. EW contributed to the data from her research study that is associated with the LA research. RF assisted with the translation of the French transcripts. BT assisted with the translation of the French transcripts and helped facilitate the live administration. All authors contributed to the article and approved the submitted version.

## Conflict of Interest

The authors declare that the research was conducted in the absence of any commercial or financial relationships that could be construed as a potential conflict of interest.

## Footnotes

1 https://canada-news.org

2 https://soundofthemuse.com/magic-mirror
