# Supplementary material for: Building Resilience During COVID-19: Recommendations for Adapting the DREAM Program – Live Edition to an Online-Live Hybrid Model for In-Person and Virtual Classrooms
Source: Front Psychol. 2021 Jul 12;12:647420. doi: 10.3389/fpsyg.2021.647420 (PMC8311233; doi:10.3389/fpsyg.2021.647420)
Supplement: Supplementary file 1 [file Table_1.DOCX]

# **Appendix A**

**DREAM Unit Summary**

Unit 1 – Mental Health & Gifted Literacy:

- Research demonstrates that young people are less open to receiving mental health promotion information if they have poor mental health literacy. Therefore, the following unit aims to enhance mental health literacy:
- Address common mental health myths and through true/false game
- Common mental health symptoms (song), difference between fear vs. phobia, everyday feelings vs. depression, who kids can turn to for help
- Gifted literacy – typical for bright children to experience strong feelings  they’ll learn tools in the program that can help them manage strong feelings

Unit 2 – Emotion Recognition & Social-Emotional Literacy:

- Six universal feelings that everyone in the world shows on their faces  discussion: What feelings do they think everyone in the world shows?
- Song about universal emotions and how two people can feel differently in the same situation
- Story to illustrate why two people might feel differently in the same situation (they have different thoughts or actions in response to the situation)
- “Emotions Go Fish” Game – cards printed from internet. Have the children in groups play “Emotions Go Fish” (played just like regular Go Fish; e.g., Do you have “sad?” Yes! Or Go Fish). When matched pairs of emotions are collected (aside from pairs in the initial hand of 5 dealt), the child who collected the pair talks about a situation in which kids in general might feel that feeling (on the matched cards). The rest of the group members then discuss why someone might feel that way. They then talk about how someone might feel differently, what they might feel, and why they might feel that way in that same situation. The game ends when everyone has had a turn. (If run with a large class, have a small group of 4 to 6 students come to the front to be the ones to ask each other “Do you have sad” and discuss a situation in which someone might generally experience that feeling, but the whole class discusses how someone might feel differently in the same situation and why).
- Follow-on printable worksheet with scenarios for homework or in-class  common scenarios gifted children face, come up with a feeling and alternative feeling that children might have in these situations and why they might have these feelings.

Unit 3 – Relaxation:

- Body awareness of feelings  in-class drama or video-presented: Show me what 25% anger would look like, what would 50% anger look like, what would 100% anger look like – where do they feel 100% anger in their body as they demonstrate it? Now show me 5% anger – where do they feel that in their body? Next time they start to feel a bit sad, worried, or angry, notice where they start to feel it in their bodies so they can maybe do something to prevent it.
- Pink elephant activity (led by video) to show why relaxation works to help calm children when they are sad angry to scared
- Balloon demonstration (video-based in case of latex allergy) to illustrate diaphragmatic—belly breathing—specifically
- Everyone gets a bubbles container – deep breath for count of 4 filling up bellies like a balloon, hold breath for count of 7, breathe out for 8, letting out a long string of bubbles. If the group leader cannot find bubble multi-packs at the local dollar store, children can imagine that they have a bubble blower. Do this activity along with the song.
- Take-home or in-class activity: When children notice that they’re starting to feel sad, angry, or worried in their bodies at school or home, try the 4-7-8 breathing a few times. After they do this breathing, colour their before/after feelings on the hand-out to notice how they can have an impact on their own feelings.

Unit 4 – More Calm Down Activities: Worry Time, Imagery, Humour

- Song – what is it teaching? How to create a worry time and how to create an imagery scenario to feel a bit better.
- Imagery activity – Draw the most relaxing scene that they can imagine. Imagine the scene using as many senses as they can (why they would see, hear, touch, smell, and maybe even taste in this scene). Video discussion – how imagery can be helpful.
- Class activity – spaghetti dance (led by video). Purpose: Show how humour or doing something funny can be relaxing.
- Class activity – progressive muscle relaxation  robot, jellyfish led by video
- At home/In-class activity  continue using the 4-7-8 breathing, add in these other activities, colour the before-after faces to see which ones are most helpful for each child (they get to keep this themselves, for their own reference to know what’s helpful for them).

Unit 5 – Avoidance & Obsessive Behaviours

- Drama activity 🡪 what happens to fears when we avoid things? (Can be shown on video or done in class) – kids pretend to be people or a dog, the scenario involves avoiding dogs because of a bite vs. not avoiding dogs after the bite – what happens to fear in each scenario?
- Song about avoidance and physical symptoms 🡪 how physical symptoms can be the first sign of stress/anxiety
- Popsicle stick craft 🡪 talking back to the mosquito (or whatever other pest the children come up with) 🡪 ties into obsessions or gifted perfectionistic behaviours

Unit 6 – Enjoyable Distraction

- Song – how enjoyable distraction/helpful thinking can help children calm down
- Group game – either use 3 balloons, or if there is a latex allergy in the school, use balls 🡪 video tells the children the story about a bad day and has the children imagine that they had a bad day like that. How do they feel now that they’re thinking about that bad day (0 – feel bad to 10 – feel great). Try to keep the balloons/balls up for 1 minute, if they drop them, that’s OK. How do they feel now? 0 to 10 🡪 they’ll feel better!
- Generating helpful list of distraction activities 🡪 class comes up with list of short, fun activities that they could use to feel less sad, angry, scared, or stressed. This isn’t avoidance, it’s just helping them feel a bit better/calm down so they can think more clearly about things.

Unit 7 – Meaningful Living

- Children make a card for someone they appreciate. Group discussion during craft: How do they think the person (or animal, as a number of children in our past groups made a card for their pet) will feel when they give them the card. How will they themselves feel when they give the card? Video: When we do things for others, we feel good ourselves.
- Class discussion/brainstorm (continued during craft): What sort of things could they do in their house to make a difference and feel good themselves? What could they do at school to make a difference? Is there a problem in the world that bothers them (environment, bullying, etc.)? Brainstorm things they could do to solve this problem in even a small way.
- Youth Engagement song – regular engagement in enjoyable activities also help us to feel good.
- At home/in-class activity – circle activities on the fun engagement catalogue that they might enjoy doing at school, at home, or in the community regularly

Unit 8 – Connection Between Thoughts & Feelings

- Video story about the connection between thoughts and feelings
- Song that tells a story about a boy whose thoughts got in the way of his running performance 🡪 what thoughts led to the boy’s tricky feelings? What thought helped him win?
- Crown hand-out 🡪 children cut out and decorate crowns (or facilitator could just pre-make crowns to save time). Cut out the print-out of thoughts online and stick one “stinky thought” to each crown. Scenarios are relevant to gifted experiences (oversensitivities, perfectionism, common worries, particular challenges with peers, etc.). Make the feeling face that goes along with the scenario. Thoughts cause feelings.
- Take-home/in-class solo practice worksheet: Common stinky thoughts for gifted children. Write down the feeling someone might have if they have this thought.

Unit 9 – Choosing to Think Differently

- Thought detective song – feelings as important “alarm bells” like a fire alarm that can tell us we’re having a stinky thought. Remind them about emotions go fish and how people can have the same thing happen, but if they think or act differently, they can feel differently. Remind them how they can use their relaxation tools to feel differently.
- Group activity – Use song verses to figure out the stinky thought and feelings that the characters in the song are having and help the characters come up with more helpful thoughts, good reasons for these more helpful thoughts, and the new feelings that the characters might have with the more helpful thoughts
- Divide the class into pairs. Give each pair two stinky thoughts from Unit 8. Each person hides their thought (taking turns). In pairs, play “hot and cold” as “thought detectives” to try to find the hidden thoughts around the room. Once they find the thoughts, discuss more helpful thoughts a person could have in the same situation, reasons for these thoughts, and how the person might feel if they have these helpful thoughts.
- At-home/In-Class – practice scenarios.

Unit 10 – “Act as If,” Helpful Problem-Solving, and Putting it All Together

- Song about becoming who we want to be
- Story building on the song about becoming who we want to be
- Superhero group activity based on song/story – come up with things that an Anger Shaker Superhero (someone who can control their anger), Joy Waker Superhero (someone who knows how to make themselves feel a little bit better if they’re sad or see the joy in everyday life), Fear Breaker Superhero (someone who can confront their fears), and Peacemaker Superhero (someone who spreads kindness and creates connection between others) can do—or think—to be a superhero. Use strategies learned to date in this discussion.
- Problem-Solving Comics – based on the superhero discussion: Draw the feeling face, the thoughts, and ways to solve the problem (helpful thinking, relaxation, engagement, etc.).

# **Appendix B**

**Ethics Approval**

# **Appendix C**

**Focus Group Guide for School Board Staff**

1)     **Acceptability.**

- Which content should be strictly delivered as information provided via video vs. which components are teachers comfortable with for rich in-class discussions?
- In the 1980s, classrooms implemented an assault prevention program delivered through video with group-based activity components to accompany the video. The video from the 1980s program looked something like this:<https://binged.it/2q2YbG0> Is this format/delivery style still relevant to today’s children (ages 6 to 10)? What delivery differences would staff suggest to make it more relevant to their students’ needs?
- Which activities are teachers comfortable with administered live in the classroom vs. viewed in an online video?
- What would facilitate classroom delivery of the program from a time, resource, and ease of administration perspective?
- Do you have any suggestions for a user-friendly web interface allowing the program to be delivered to the whole class at once?
- How do staff visualize child-friendly "music video" delivery of some program information? What would make the music videos “cool” to kids?
- To ensure quality and program success, if program evaluation is built in to the online interface, what would facilitate pre-post brief survey completion online? In our pilot study, children either took turns at their leisure clicking on video-based items on computers with headphones in settings in which the program was delivered (pre-post program administration) or completed them at home (pre-post program administration). Sample evaluation survey which could be built into the web-interface:<https://www.surveymonkey.ca/r/RL5TC6D> - pilot version of survey.
- Of note, 3 children currently enrolled in the OCDSB congregated gifted program (2 primary, 1 older) acted in these survey video clips, the younger 2 in the spring prior to September 2016 CGP program entry.
- What would enhance teacher/school board interest in program uptake?
- When gifted children aren't participating within a congregated gifted classroom, how might gifted children in a regular classroom be grouped together for program administration?

2)     **Feasibility**.

- Is the proposed online-live hybrid program perceived to be feasible to use within classroom and community group settings?
- Are there suggestions to make the program more feasible from a time and resource perspective? For example, how should units be grouped together to facilitate program administration, balanced with other classroom demands?
- In community groups, we ran the full program in three 1.5h sessions? Might the program be best delivered in a classroom in the individual 10-unit (5 to 30 minutes each) format or is there a particular time chunking that would be better?

3)     **Sustainability**.

- Does the proposed online D.R.E.A.M. Program appear to be sustainable for use by organizations?

4)     **Credibility.**

- Do teachers/school board staff perceive that the hybrid online-live program would lead to the following desired outcomes: Reduced perfectionism, enhanced openness to learning, emotion regulation, good problem-solving skills, meaningful engagement in the community, and enhanced socio-emotional literacy relevant to gifted children.

# **Appendix D**

**Satisfaction Survey Items**

1. **How happy were you with the DREAM program (songs, what you learned, what we did)? Circle the person on the tree**

**
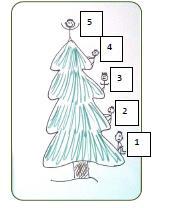
5[top of tree] = happy**

**4 = mostly happy**

**3 = so-so**

**2 = mostly unhappy**

**1[bottom of tree] = unhappy**

**What did you like? Do you have ideas to make this program better:**

**______________________________________________**

**______________________________________________**

**______________________________________________**

- **Do you think this program gives people “tools” to help them be more in control of their feelings (e.g., through healthy thinking, mindful relaxation, doing enjoyable activities to feel better, and problem solving)? Circle the person on the tree:**

**
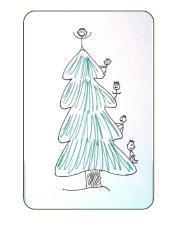
5 (top of tree) = yes**

**4 = probably yes**

**3 = so-so**

**2 = probably not**

**1 = no**

**Do you think this program might help people feel better about themselves? Circle the person on the tree:**

**
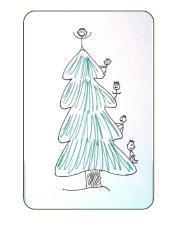
**

**5 (top of tree) = yes**

**4 = probably yes**

**3 = so-so**

**2 = probably not**

**1 = no**

- **Do you think this program helps people feel more “connected” to the other kids, to their family, or to their community? Circle the person on the tree:**

**5 (top of tree) = yes**

**
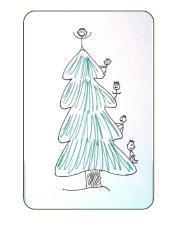
**

**4 = probably yes**

**3 = so-so**

**2 = probably not**

**1 = no**

- **Do you think this program helps people to have more courage to try tricky or other new things? Circle the person on the tree:**

**
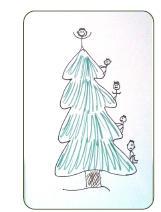
**

**5 (top of tree) = yes**

**4 = probably yes**

**3 = so-so**

**2 = probably not**

**1 = no**

- **Do you think this program helps people feel hopeful about the future and what they can do? Circle the person on the tree:**

**5 (top of tree) = yes**

**4 = probably yes**

**3 = so-so**

1. **= probably not**

**1= no**

1. **If the DREAM program were to be presented in video clips online at home to go along with print-off activities to do together at home (i.e. the activities you did here), what would be the best length for each teaching video clip, music video, and activity combined (e.g., 5 minutes, 10 minutes, 15 minutes)? Why?**

**___________________________________________________________________**

**8) Participation Level:**

**Passive: Do you want to watch videos to learn? or Active: Do you want to do activities and talk as a family? Circle the number that fits the amount of passive vs. active activities that you want for this program.**

**Passive 0__1__2__3__4__5__6__7__8__9__10 Active**

1. **Would you like this program if it were taught to you through videos on the computer, but you still got to do the other activities? (Circle) Yes/No. If no, why not:**

**___________________________________________________________________**

**___________________________________________________________________**

1. **What parts of the program did you like the best? In other words, what should we keep the same if we put the program online?**

**___________________________________________________________________**

**___________________________________________________________________**

1. **What parts of the program did you like the least? In other words, what should we change if we put the program online?**

**___________________________________________________________________**

**___________________________________________________________________**

1. **For children and families, what parts of the program do you think will help you to remember to use the things that you learned?**

**___________________________________________________________________**

**___________________________________________________________________**

**___________________________________________________________________**

1. **Do you have any suggestions for things that we could add to the program online to help you to remember to use the things that you learned?**

**___________________________________________________________________**

**___________________________________________________________________**

**___________________________________________________________________**

1. **The short-term goals of this program are to: increase dialogue and knowledge around mental health and reduce stigma, increase well-being and meaning, and build a toolbox of skills:**

**a) Does the program seem to do these things? (Circle) Yes/No. Why? Or Why Not?**

**___________________________________________________________________**

**___________________________________________________________________**

**b) What could we do better to meet these goals?**

**___________________________________________________________________**

**___________________________________________________________________**

1. **Do you believe that online videos with printable discussion topics and activities could help achieve these goals? (Circle) Yes/No. Please briefly explain your answer.**

**___________________________________________________________________**

**___________________________________________________________________**

**___________________________________________________________________**

**___________________________________________________________________**

# **Appendix E**

**DREAM Waitlist Focus Group Questions**

1. Hearing your concerns about screen time- would you recommend airing more on the live discussion side versus the tv/ computer?
2. If the children were to come home with homework to do with the family could you see yourself engaging with that?

# **Appendix F**

**Waitlist Focus Group Consent**

Dreaming of a Solution: D.R.E.A.M-O.F. an Online Mental Health Promotion Program for Children and Their Families on Mental Health Waitlists

Purpose of the Study

Emmalyne Watt, Ph.D candidate in Counselling and Spirituality at Saint Paul University, supervised by Dr. Laura Armstrong, is carrying out a research study examining whether families on mental health waitlists could benefit from a brief mental health promotion program prior to accessing standard mental health services. Key implications of the study involve examining whether promoting resilience and meaning, enhancing family quality of life and family resilience will ultimately result in a reduction of childhood mental health symptoms and, potentially, shorten service time needed.

Procedure

If you and your family agree to take part in the study, you will be asked to participate in a focus group where you will be presented with a series of questions. Your responses to these questions will help us to incorporate the lived experiences and needs of families directly into our program in order to better meet your needs. The focus group will be auditory-recorded so that the content may be further referenced for accuracy. Following the completion of the focus group, at a later date, you will be invited to participate in the family mental health promotion program.

If you or your family members feel uncomfortable answering some of these personal questions, then you or they may refrain from doing so. Participation in the focus group may take approximately 60 minutes of your family’s time.

Rights of Participants

If you and your family decide to participate, you will be free to withdraw from the study at any time. In addition, you are free to refuse to answer any question during the focus group or on the questionnaires. Participation in the study is fully voluntary. The information that your family provides will help greatly in our understanding of family meaning and resilience as it related to reducing childhood mental health symptoms and enhancing family coping. All information collected from your family will remain completely confidential and will be stored in a locked office on an encrypted, password protected computer. Questionnaires will be number or letter coded and anonymous. Answers will remain confidential and will be used for research purposes only. If consent for participation is withdrawn, then your family’s data will not be included in our analyses and will be securely deleted.

Limits to Confidentiality and Benefits of Participation

Although participation risk is minimal, anonymity isn't guaranteed due to the nature of group-meeting. However, participants may benefit from meeting others experiencing similar concerns. For research purposes, anonymity is guaranteed in all publications, as data will be number coded.

Furthermore your son/daughter will have an opportunity to participate in a program that has been shown to promote resilience, reducing internalizing and externalizing symptoms of childhood mental illness. Given this, participation may be beneficial for your child. The addition of family focused strategies are new in the current research. This program, therefore, will hopefully also be beneficial for the whole family. Participation in the pre-group and post-group questionnaires may also be fun for children, as they get to watch brief video clips.

Contact Information

This research has been reviewed by the Saint Paul Research Ethics Committee. This committee helps ensure and protect the rights and welfare of those participating in research. If you have any other concerns or questions, they can be directed to Dr. Laura Armstrong at ***-***-****, ext. ****. The chair of research and ethics can also be reached at ***-***-****.

Please sign below to provide your consent and the consent of your child to participate.

# **Appendix G**

**School Board Focus Group Consent**

"All information collected will be used to develop recommendations for the creation of the online version of our program. Everything that we discuss will be written down anonymously, so your names will not appear in the data collected. You can choose to answer or not answer any questions asked about the design of the program. You're also welcome to leave at any point in time before the end of the meeting or at the end. Do you have any questions?"

# **Appendix H**

**Live Administration, Pre and Post-Test Consent**

Satisfaction Survey Consent Dreaming of a Solution: D.R.E.A.M-O.F. an Online Mental Health Promotion Program for Children and Their Families on Mental Health Waitlists

We are delivering a socioemotional educational program for families in Ottawa in Winter 2019. DREAM (Developing Resilience through Emotions, Attitudes, and Meaning) is a program developed by Dr. Laura Armstrong, a Clinical Psychologist and Assistant Professor at Saint Paul University. Emmalyne Watt, Ph.D. Candidate, has further designed family units for this program (DREAM-OF).

In the program, families will learn social and emotional skills to manage worries, sadness, anger, obsessions, conflict, perfectionism, and much more. The goal is to have fun while learning lifelong, evidence-based skills through games, music, drama, and crafts.

This is the first year that we have offered the family version of this program, so we ask that you and your children complete our pre-group and post-group questionnaires. We will use these questionnaires to measure what the program is doing well and what we can improve upon to refine the program. Ms. Watt will also use the data to complete her doctoral dissertation.

If you agree to participate in our pre and post group questionnaires, please ask your child to complete the 2 online links, which relate to skills taught in the program, both before and after your child takes part in the program activities. Parents have 5 questionnaires to complete. Participation in the surveys will take approximately 20-25 minutes of your child's time.

Rights of Participants

If you and your family decide to participate, you will be free to withdraw from the study at any time. In addition, you are free to refuse to answer any questions on the questionnaires. Participation in the study is fully voluntary. The information that your family provides will help greatly in our understanding of family meaning and resilience as it related to reducing childhood mental health symptoms and enhancing family coping. Although we initially ask you to put your names on questionnaires, our research assistant will only use this to match parent and child data, as well as pre and post group data. These names will then be removed and number-coded. Therefore, all information retained from your family will become completely confidential and will be stored in a locked office on an encrypted, password protected computer. If consent for participation is withdrawn, then your family’s data will not be included in our analyses and will be securely deleted.

Limits to Confidentiality and Benefits of Participation

Although participation risk is minimal, anonymity isn't guaranteed due to the nature of group-meeting. However, participants may benefit from meeting others experiencing similar concerns. For research purposes, anonymity is guaranteed in all publications, as data will be number coded.

Participating in this program could promote resilience, reduce internalizing and externalizing symptoms of childhood mental illness. Given this, participation may be beneficial for your child. The addition of family-focused strategies are new in the current research. This program, therefore, will hopefully also be beneficial for the whole family. Participation in the pre-group and post-group questionnaires may also be fun for children, as they get to watch brief video clips.

Contact Information

This research has been reviewed by the Saint Paul Research Ethics Committee. This committee helps ensure and protect the rights and welfare of those participating in research. If you have any other concerns or questions, they can be directed to Dr. Laura Armstrong at ***-***-****, ext. ****. The chair of research and ethics can also be reached at ***-***-****.

Sincerely,

Emmalyne Watt, Ph.D. Candidate

Question Title

* 1. Please click here if your family consents to participate

Yes

No
